# Prognostic value of central venous-to-arterial carbon dioxide difference in patients with bloodstream infection

**DOI:** 10.7150/ijms.51447

**Published:** 2021-01-01

**Authors:** Zhonghua Wang, Xuebiao Wei, Tiehe Qin, Shenglong Chen, Xiaolong Liao, Weixin Guo, Peihang Hu, Yan Wu, Jie Li, Youwan Liao, Shouhong Wang

**Affiliations:** 1 Department of Critical Care Medicine, Guangdong Geriatrics Institute, Guangdong Provincial People's Hospital, Guangdong Academy of Medical Sciences, Guangzhou 510080, Guangdong, China.; 2 Department of Critical Care Medicine, Guangdong Provincial People's Hospital, Guangdong Academy of Medical Sciences, Guangzhou 510080, Guangdong, China.; Zhonghua Wang and Xuebiao Wei are contributed equally to this work as Co-first authors.

**Keywords:** central venous-to-arterial carbon dioxide difference, biomarker, prognostic factor, bloodstream infection

## Abstract

**Background:** Bloodstream infection (BSI) are prone to circulation disorders, which portend poor outcome. The central venous-to-arterial carbon dioxide difference (Pcv-aCO_2_) is a biomarker for circulation disorders, but the prognostic value of Pcv-aCO_2_ in BSI patients remains unclear. This study was to investigate the association of Pcv-aCO_2_ with adverse events in BSI patients.

**Methods:** The patients with BSI between August 2014 and August 2017 were prospectively enrolled. Clinical characteristic and laboratory results were collected. We analyzed the association of the level of Pcv-aCO_2_ with clinical variables and 28-day mortality.

**Results:** A total of 152 patients were enrolled. The Pcv-aCO_2_ was positively correlated with white blood cell count (r=0.241, p=0.003), procalcitonin (r=0.471, p<0.001), C-reactive protein (r=0.192, p=0.018), lactate (r=0.179, p=0.027), Sequential Organ Failure Assessment (r=0.318, p<0.001) and Acute Physiology And Chronic Health Evaluation II score (r=0.377, p<0.001), while that was negatively correlated with central venous oxygen saturation (r=-0.242, p<0.001) and platelet (r=-0.205, p=0.011). Kaplan-Meier curves demonstrated that patients with Pcv-aCO_2_ >6mmHg had a worse prognosis than those without (log rank=32.10, p<0.001). Multivariate analysis showed Level of Pcv-aCO_2_ was an independent risk factor for 28-day mortality (HR: 3.10, 95% CI: 1.43-6.74, p=0.004). The area under the receiver operating characteristic curve of Pcv-aCO_2_ for prediction of 28-day mortality in patients with BSI was 0.794. Pcv-aCO_2_>6 mmHg had 81.1% sensitivity and 78.8% specificity for predicting 28-day mortality.

**Conclusion:** Pcv-aCO_2_ may be a simple and valuable biomarker to assessment of 28-day mortality in patients with BSI.

## Introduction

Bloodstream infection (BSI) is common systemic infection in intensive care unit (ICU), affecting 189/100000 patients 1. It is associated with increased risk of organ failure and mortality, and one-year mortality from BSI is between 8% and 48% 2. BSI bring a huge financial burden to families and society 3. Therefore, early accurate identification of patients at high risk of poor outcomes may have an essential role in improving prognosis.

The common pathological change of BSI is microcirculation disorder 4. Previously, lactate and central venous oxygen saturation (S_CV_O_2_) are accepted clinical indicators of organ perfusion and oxygen metabolism 5, 6. At present, central venous-to-arterial carbon dioxide difference (Pcv-aCO_2_) as a biomarker of perfusion is gradually recognized. Pcv-aCO_2_ has reported to be associated with poor prognosis in critical patients suffering from shock, and Pcv-aCO_2_ is recommended as a biomarker for further resuscitation interventions 7. In addition, persistently high Pcv-aCO_2_ during the early phases of resuscitation was a predictor for poor outcomes in patients with septic shock 8, 9. However, the prognostic role of Pcv-aCO_2_ in patients with BSI remains to be seen. In this study, we attempted to explore the association between Pcv-aCO_2_ and 28-day mortality in patients with BSI.

## Patients and methods

### Study objects

We prospectively enrolled patients with BSI between August 2014 and August 2017 in Geriatric ICU, Guangdong Provincial People's Hospital, Guangzhou, China. Inclusion criterion were as follows: (1) age>18 years; (2) clinical symptoms meet systemic inflammatory response syndrome or hypotension; (3) at least one positive blood culture; (4) only a single bloodstream infection pathogen. Exclusion criteria included: (1) patients refused cardiopulmonary resuscitation or aggressive measures; (2) mismatch of the time of arterial and venous blood gas test. The eligible patients were divided into two groups based on a cut-off value of Pcv-aCO_2_ of >6mmHg from previous study on septic shock 8. The present study was approved by the Ethics Committee of Guangdong Provincial People's Hospital (No. GDREC2014006H), and written informed consent was obtained from all included patients or their relatives.

### Data collection

The demographic including sex, age were recorded and Acute Physiology And Chronic Health Evaluation II (APACHE II) score, and Sequential Organ Failure Assessment (SOFA) were calculated when the patients were diagnosed with BSI. The biochemical data, such as Pcv-aCO_2_, ScvO_2_, white blood cell (WBC) count, platelet (PLT), procalcitonin (PCT), C-reactive protein (CRP), and lactate were tested when the patients were diagnosed with BSI. We routinely performed arterial and central venous blood gas analysis while taking blood culture specimens. Pcv-aCO_2_ and ScvO_2_ were obtained by simultaneous analysis of arterial and central venous blood gases with a blood gas analyzer (ABL 800; Radiometer Medical, Denmark); other demographic and clinical characteristics of enrolled participants were collected by a researcher with the use of an electronic case report form, and then were confirmed by another researcher.

### Follow-up and endpoints

All the patients were followed-up for 28 days by telephone interviews after BSI diagnosis. The primary endpoint was 28-day all-cause mortality.

### Statistical analysis

Statistical analyses were performed by using SPSS 24.0 software (IBM, Armonk, NY, USA). The continuous variables were presented as mean ± standard deviation (SD), and compared using independent sample t-test when they were normally distributed; for non-normally distribution, the Wilcoxon rank-sum test was conducted, and the data presented as median and interquartile range. Categorical variables were presented as a percentage and compared using χ^2^ or Fisher's exact test. Bivariate correlations were calculated by Pearson's or Spearman's correlation coefficients. Survival curves were depicted by using Kaplan-Meier analysis. Cox proportional hazards regression model was used to analyze the association between the variables and the risk of death. Multivariate Cox regression analysis was performed with the variables whose p-value was less than 0.05 in univariate logistic regression analysis for 28-day mortality. Receiver operating characteristic (ROC) curves were plotted to evaluate the predictive power of Pcv-aCO_2_ for 28-day mortality. A p-value < 0.05 was considered statistically significant.

## Results

### Clinicopathological characteristics of patients with BSI

Finally, 152 patients were enrolled and 53 (34.9%) patients died within 28 days. All the patients were divided into two groups: Pcv-aCO_2_ ≤ 6 mmHg (n=88) and Pcv-aCO_2_ > 6 mmHg (n=64). The baseline characteristics were presented in **Table 1**. There were no significant differences between the two groups in terms of age, gender, concurrent foci of infection, the main history, non-infection comorbidity and CRP. The SOFA (11.89 ± 4.08 vs 9.69 ± 3.18, p<0.001), APACHE II score (28.63 ± 6.11 vs 24.78 ± 5.38, p<0.001), PCT (34.17 ± 39.52 vs 9.73 ± 21.24, p=0.001), WBC (16.90 ± 7.12 vs 14.20 ± 6.02, p=0.013), lactate (3.48 ± 3.44 vs 2.34 ± 2.67, p=0.023) were higher and platelet (114.78 ± 80.42 vs 146.23 ± 90.05, p<0.028) , ScvO_2_ (53.15 ± 12.05 vs 70.53 ± 10.73, p<0.001) were lower in Pcv-aCO_2_ > 6 mmHg group. The demand for mechanical ventilation, vasopressor and continuous renal replacement therapy (CRRT) in Pcv-aCO_2_ > 6 mmHg group is significantly greater than in Pcv-aCO_2_ ≤ 6 mmHg group. Gram-positive (GP) bacteria were the predominant pathogens in Pcv-aCO_2_ ≤ 6 mmHg group, while Gram-negative (GN) bacteria are the predominant pathogens in Pcv-aCO_2_ > 6 mmHg group. The 28-day mortality (60.9% vs 15.9%, p<0.001) was higher in Pcv-aCO_2_ > 6 mmHg group.

### Correlation of Pcv-aCO_2_ with other parameters

We found that Pcv-aCO_2_ was positively correlated with WBC (r=0.241, p=0.003), PCT (r=0.471, p<0.001), CRP (r**=**0.192**,** p=0.018**)**, lactate (r**=**0.179**,** p**=**0.027**)**, SOFA (r=0.318, P<0.001), and APACHE II score (r**=**0.377, p<0.001), while that was negatively correlated with Sc_V_O_2_ (r** =**-0.242, p<0.001) and PLT (r =-0.205, p=0.011) (**Table 2**).

### Pcv-aCO_2_ and 28-day mortality

**Figure 1:**
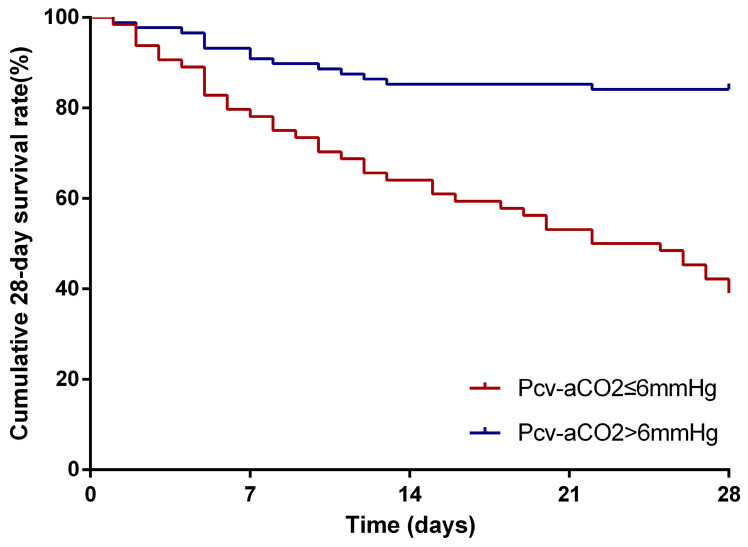
Kaplan-Meier analysis for depicting the curves of the 28-day cumulative survival rates. Comparing patients in the groups of Pcv-aCO_2_ ≤ 6 mmHg and Pcv-aCO_2_>6 mmHg showed that the 28-day survival rates were significantly lower in patients with Pcv-aCO_2_>6 mmHg than those with Pcv-aCO_2_ ≤ 6 mmHg (p<0.001)

The survival curves were shown in **Figure 1**. Kaplan-Meier survival curves show that patients with Pcv-aCO_2_ > 6 mmHg had a worse prognosis than those with Pcv-aCO_2_ ≤6 mmHg (log-rank=32.10, p< 0.001).

The risk of death was significantly correlated with the levels of Pcv-aCO_2_ (hazard ratio (HR): 4.90, 95% confidence interval (CI): 2.66-9.05, p<0.001), PLT (HR: 3.90, 95% CI: 2.16-7.03, p<0.001), PCT (HR: 2.87, 95% CI: 1.62-5.07, p<0.001), lactate (HR: 2.53, 95% CI: 1.46-4.45, p=0.001), Sc_V_O_2_ (HR: 2.04, 95% CI: 1.17-3.53, p=0.011), SOFA (HR: 3.25, 95% CI: 1.81-5.86, p<0.001) and APACHE Ⅱ score (HR: 2.61, 95% CI: 1.44-4.75, p=0.002) . Multivariate Cox regression analysis was used to analyze the associations of the risk of death and adjustment of Pcv-aCO_2_, PLT, PCT, lactate, Sc_V_O_2,_ SOFA and APACHE Ⅱ score. The levels of Pcv-aCO_2_ (HR: 3.10, 95% CI: 1.43-6.74, p=0.004) and PLT (HR: 2.08, 95% CI: 1.08-3.98, p=0.028) were independent risk factors for 28-day mortality in patients with BSI (**Table 3**).

### The predictive accuracy for 28-day mortality

**Figure 2:**
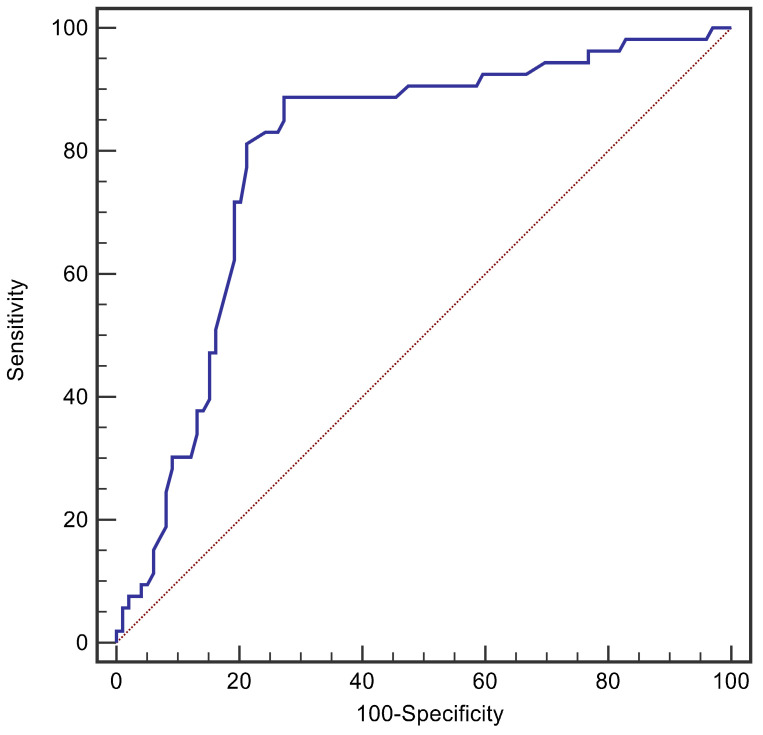
The receiver operating characteristic curves for Pcv-aCO_2_ levels in predicting 28-day mortality of the patients with BSI.

The ROC curve of Pcv-aCO_2_ predicting 28-day mortality in patients with BSI, as shown in **Figure 2**, revealed that the area under the ROC curve was 0.794. Furthermore, Pcv-aCO_2_ greater than 6 mmHg was the best threshold for predicting 28-day mortality, with a sensitivity of 81.1% and specificity of 78.8%.

## Discussion

The present study demonstrated that increased Pcv-aCO_2_ at the time of blood culture was independently associated with 28-day mortality in patients with BSI. In addition, Pcv-aCO_2_>6 mmHg was a valuable predictor of the increased risk of 28-day mortality. In this study, the 28-day mortality was 34.9%, which were higher than previous studies 10. It might be due to the advanced age of the patients in our study, because age is one of the risk factors for poor prognosis 11. The other reasons should be considered was more serious patients enrolled in our study. Patients included in this study had higher SOFA and APACHE II score than previous studies 10.

It is well-known that BSI often cause circulatory disorder, which associated with poor outcomes 12. In recent years, Pcv-aCO_2_ is considered one of indicator for monitoring of tissue perfusion 13. A previous study reported that a significant increase in Pcv-aCO_2_ during cardiac arrest results from cardiovascular stagnation 14. Similarly, another research showed that Pcv-aCO_2_ is inversely proportional to cardiac output in animal models of hemorrhage, hypovolemia, and obstructive shock 15-17. When cardiac output reduce, the blood flow is slow and the elution ability of CO_2_ is impaired; therefore, more CO_2_ is accumulated in the tissue, and the Pcv-aCO_2_ increases as the CO_2_ diffuses easily and quickly 18. In the early fluid resuscitation of septic shock, a persistent increase in Pcv-aCO_2_ suggests a poor prognosis, and monitoring Pcv-aCO_2_ during early fluid resuscitation is a helpful indicator for assessing the adequacy of tissue perfusion 8, 19. As an indicator of tissue perfusion, Lactate is the goal of early fluid resuscitation in septic shock 20. There was a correlation between Pcv-aCO_2_ and lactate in our study. In Pcv-aCO_2_ > 6 mmHg group, the lactate level, circulatory disorders and vasopressor used were more, which suggest that Pcv-aCO_2_ can be an good indicator of tissue perfusion and prognosis.

In severe infections, oxygen supply-demand imbalance often leads to increased mortality 21. In fact, Pcv-aCO_2_ monitoring not only can reflect cardiac output and tissue microcirculation perfusion, but also show a balance between tissue oxygen supply and demand, thereby objectively reflecting tissue oxygen metabolism 22. A study showed that the changes of cardiac output were not consistent with those of Pcv-aCO_2_ during septic shock, suggesting that hemodynamic changes cannot be used to explain an increase in Pcv-aCO_2_ in this case, which may be associated with poor tissue oxygen supply and increased oxygen consumption 23. Kocsi *et al* 24 found that Pcv-aCO_2_ is an important indicator to monitor an imbalance between tissue oxygen supply and demand caused by low blood volume. The oxygen supply and demand of tissue can be timely detected by monitoring dynamic changes of Pcv-aCO_2_ in case of insufficient blood volume.The ratio of Pcv-aCO_2_ to arteriovenous oxygen contentis difference is a better marker of global anaerobic metabolism than lactate in septic shock patients 25. Furthermore, we found that Pcv-aCO_2_ was negatively correlated with S_CV_O_2_ which is an indicator of the balance of oxygen metabolism, suggesting that Pcv-aCO_2_ is an appropriate indicator for tissue perfusion and oxygen metabolism.

The mortality of bloodstream infections caused by different pathogen is different 26. Furthermore, different pathogen infections may cause different circulation disorders 27, which indicated by Pcv-aCO_2_. Since Pcv-aCO_2_ can reflect tissue perfusion, it may be used for the identification of BSI pathogens. As we know, PCT plays an important role in the diagnosis of BSI 28. It helps identify pathogens and guides the choice of antibiotics 29. In this study, Pcv-aCO_2_ and PCT were found to be significantly correlated, suggesting that Pcv-aCO_2_ may contribute to the identification of pathogens. Moreover, we found the pathogen distribution was different in different groups presented based on the level of Pcv-aCO_2_, and there was more GN bacteria infection in the higher level of Pcv-aCO_2_ group. This shows that Pcv-aCO_2_ can provide a clue to identify pathogens and predict outcomes.

Additionally, we found that Pcv-aCO_2_ has a significant correlation with S_CV_O_2_, lactate, PCT, CRP, SOFA and APACHE II score in patients with BSI. PCT and CRP plays an important role in the early diagnosis and prognosis of BSI patients 30, 31. S_CV_O_2_ and lactate guides early fluid resuscitation can effectively predict the prognosis of patients with sepsis 32, 33. Pcv-aCO_2_, S_CV_O_2,_ and lactate are taken as important indicators for treatment and prognosis of sepsis 34. In our study, the patients with different levels of Pcv-aCO_2_ showed significant differences in S_CV_O_2_, lactate, PCT, PLT, SOFA, APACHE II score, and prognosis. The use of mechanical ventilation, vasopressor, and CRRT were significantly more in Pcv-aCO_2_ >6mmHg group than those in Pcv-aCO_2_ ≤6mmHg group. Therefore, Pcv-aCO_2_ can properly reflect the illness severity of the patient. We also further analyzed the association between Pcv-aCO_2_ and the risk of death by using Cox regression analysis, which indicated that Pcv-aCO_2_ was an independent risk factors for 28-day mortality. After that, ROC curves were plotted to evaluate the predictive power of Pcv-aCO_2_ for the occurrence of 28-day mortality. We found that the Pcv-aCO_2_ is an important predictive factor for 28-day mortality in patients with BSI.

### Limitation

This was a retrospective analysis based on prospectively collected data. There are several limitations in this study. One limitation is that there was no dynamic monitoring of Pcv-aCO_2_, that may ignore the impact of different treatments on clinical outcomes. In addition, the use of basic drugs, especially sodium bicarbonate, in patients during the study was not fully clarified, which may affect the accuracy of the results of Pcv-aCO_2_ monitoring. Moreover, this is a single-center study, that included limited research samples, therefore further multi-center large-scale studies need to be conducted.

## Conclusion

Our results demonstrated that increased Pcv-aCO_2_ while blood culture was an independent predictor of 28-day mortality in patients with BSI, even after adjusting a previous risk model. Furthermore, patients with Pcv-aCO_2_ greater than 6mmHg were more likely to have poor outcomes. The use of Pcv-aCO_2_ as a prognostic marker provides valuable information for risk stratification.

## Figures and Tables

**Table 1:** Patients' baseline clinical characteristics at different levels of Pcv-aCO_2_

Clinical variables	Pcv-aCO_2_≤6mmHg (n=88)	Pcv-aCO_2_>6mmHg (n=64)	p
Age (years)	80.55 ± 7.62	82.58 ± 8.24	0.119
Women, n (%)	31(35)	19(30)	0.200
SOFA	9.69± 3.18	11.89± 4.08	<0.001
APACHE Ⅱ	24.78± 5.38	28.63± 6.11	<0.001
Concurrent foci of infection(%)			
Pneumonia	64(72.7)	38(59.3)	0.084
Urinary tract infection	15(17.0)	10(15.6)	0.600
Abdomen infection	3(3.4)	7(10.9)	0.129
Skin and soft tissues infection	4(4.5)	7(10.9)	0.236
Others	1(1.1)	2(3.1)	0.780
History, n (%)			
Diabetes	30(34.1)	20(31.3)	0.713
Hypertension	51(57.9)	33(51.6)	0.434
Cerebral infarction	30(34.1)	15(23.4)	0.155
COPD	28(31.8)	12(18.8)	0.071
CAD	16(18.2)	7 (10.9)	0.192
Previous cardiovascular surgery	9(10.2)	6(9.4)	0.862
Chronic renal insufficiency	10(11.4)	5(7.8)	0.469
Non-infection comorbidity, n (%)			
NYHA Ⅲ-Ⅳ	21(23.9)	11(17.2)	0.319
Surgical treatment	9(10.2)	6(9.4)	0.862
Massive haemorrhage	6(6.8)	2(3.1)	0.523
Traumatic brain injury	7(8.0)	2(3.1)	0.369
PCT (ng/ml)	9.73 ± 21.24	34.17± 39.52	<0.001
WBC(x10^3^/mm^3^)	14.20 ± 6.02	16.90± 7.12	0.013
Plateles (x10^3^/mm^3^)	146.23 ± 90.05	114.78 ± 80.42	0.028
CRP(mg/L)	122.60 ± 61.37	130.35± 52.86	0.417
Lactate (mmol/L)	2.34 ± 2.67	3.48 ± 3.44	0.023
Sc_V_O_2_(%)	70.53± 10.73	53.15± 12.05	<0.001
Microbiology			
Gram-positive bacteria, n (%)	54(61.4)	21(32.8)	<0.001
Gram-negative bacteria, n (%)	15(17.0)	42(65.6)	<0.001
Fungus, n (%)	18(20.5)	2(1.6)	<0.001
Mechanic ventilation, n (%)	60(68.2)	58(90.6)	0.001
Vasopressor, n (%)	50(56.8)	50(78.1)	0.006
CRRT, n (%)	20(22.7)	32(50.0)	<0.001
28-day death (%)	14(15.9)	39(60.9)	<0.001

SOFA, Sequential Organ Failure Assessment; APACHE Ⅱ, Acute Physiology And Chronic Health Evaluation II; COPD, chronic obstructive pulmonary disease; CAD, Coronary Artery Disease; PCT, procalcitonin; WBC, white blood cell; CRP, C-reaction protein; Sc_V_O_2_, central venous oxygen saturation; CRRT, continuous renal replacement therapy; NYHA, New York Heart Association.

**Table 2:** Spearman's correlation analysis between Pcv-aCO_2_ and other clinical variables among all patients included in the study (n=152)

	r	p
WBC	0.241	0.003
Platele	-0.205	0.011
PCT	0.471	<0.001
CRP	0.192	0.018
Sc_V_O_2_	-0.242	0.003
Lactate	0.179	0.027
SOFA	0.318	<0.001
APACHE Ⅱ	0.377	<0.001

WBC, white blood cell; PCT, procalcitonin; CRP, C-reaction protein; Sc_V_O_2_, central venous oxygen saturation; SOFA, Sequential Organ Failure Assessment; APACHE Ⅱ, Acute Physiology And Chronic Health Evaluation II.

**Table 3:** Univariate and multivariate Cox regression analyses of the 28-day mortality

Variables	Univariate Cox regression			Multivariate Cox regression		
	HR	95% CI	p	HR	95% CI	p
Pcv-aCO_2_	4.90	2.66-9.05	<0.001	3.10	1.43-6.74	0.004
WBC	3.18	0.77-13.07	0.108			
Platele	3.90	2.16-7.03	<0.001	2.08	1.08-3.98	0.028
PCT	2.87	1.62-5.07	<0.001	1.39	0.72-2.69	0.327
CRP	1.40	0.81-2.43	0.229			
Sc_V_O_2_	2.04	1.17-3.53	0.011	0.72	0.38-1.36	0. 311
Lactate	2.53	1.46-4.45	0.001	1.57	0.85-2.88	0.147
SOFA	3.25	1.81-5.86	<0.001	1.86	0.91-3.82	0.89
APACHE Ⅱ	2.61	1.44-4.75	0.002	1.28	0.62-2.64	0.507

Pcv-aCO_2_, central venous-to-arterial carbon dioxide difference; WBC, white blood cell; PCT, procalcitonin; CRP, C-reaction protein; Sc_V_O_2_, central venous oxygen saturation; SOFA, Sequential Organ Failure Assessment; APACHE Ⅱ, Acute Physiology And Chronic Health Evaluation II.

## References

[1] Laupland KB (2013). Incidence of bloodstream infection: a review of population-based studies. Clinical microbiology and infection: the official publication of the European Society of Clinical Microbiology and Infectious Diseases.

[2] McNamara JF, Righi E, Wright H, Hartel GF, Harris PNA, Paterson DL (2018). Long-term morbidity and mortality following bloodstream infection: A systematic literature review. The Journal of infection.

[3] Nuckols TK, Keeler E, Morton SC, Anderson L, Doyle B, Booth M, Shanman R, Grein J, Shekelle P (2016). Economic Evaluation of Quality Improvement Interventions for Bloodstream Infections Related to Central Catheters: A Systematic Review. JAMA Intern Med.

[4] Artero A, Zaragoza R, Camarena JJ, Sancho S, Gonzalez R, Nogueira JM (2010). Prognostic factors of mortality in patients with community-acquired bloodstream infection with severe sepsis and septic shock. Journal of critical care.

[5] Chertoff J, Chisum M, Garcia B, Lascano J (2015). Lactate kinetics in sepsis and septic shock: a review of the literature and rationale for further research. Journal of intensive care.

[6] Vincent JL, De Backer D (2018). From Early Goal-Directed Therapy to Late(r) Scvo2 Checks. Chest.

[7] van Beest PA, Lont MC, Holman ND, Loef B, Kuiper MA, Boerma EC (2013). Central venous-arterial pCO(2) difference as a tool in resuscitation of septic patients. Intensive care medicine.

[8] Ospina-Tascon GA, Bautista-Rincon DF, Umana M, Tafur JD, Gutierrez A, Garcia AF, Bermudez W, Granados M, Arango-Davila C, Hernandez G (2013). Persistently high venous-to-arterial carbon dioxide differences during early resuscitation are associated with poor outcomes in septic shock. Critical care.

[9] Muller G, Mercier E, Vignon P, Henry-Lagarrigue M, Kamel T, Desachy A, Botoc V, Plantefeve G, Frat JP, Bellec F (2017). Prognostic significance of central venous-to-arterial carbon dioxide difference during the first 24 hours of septic shock in patients with and without impaired cardiac function. British journal of anaesthesia.

[10] Hattori H, Maeda M, Nagatomo Y, Takuma T, Niki Y, Naito Y, Sasaki T, Ishino K (2018). Epidemiology and risk factors for mortality in bloodstream infections: A single-center retrospective study in Japan. American journal of infection control.

[11] Kaye KS, Marchaim D, Chen TY, Baures T, Anderson DJ, Choi Y, Sloane R, Schmader KE (2014). Effect of nosocomial bloodstream infections on mortality, length of stay, and hospital costs in older adults. Journal of the American Geriatrics Society.

[12] Laupland KB, Zygun DA, Doig CJ, Bagshaw SM, Svenson LW, Fick GH (2005). One-year mortality of bloodstream infection-associated sepsis and septic shock among patients presenting to a regional critical care system. Intensive care medicine.

[13] Ospina-Tascon GA, Umana M, Bermudez WF, Bautista-Rincon DF, Valencia JD, Madrinan HJ, Hernandez G, Bruhn A, Arango-Davila C, De Backer D (2016). Can venous-to-arterial carbon dioxide differences reflect microcirculatory alterations in patients with septic shock?. Intensive care medicine.

[14] Weil MH, Rackow EC, Trevino R, Grundler W, Falk JL, Griffel MI (1986). Difference in acid-base state between venous and arterial blood during cardiopulmonary resuscitation. The New England journal of medicine.

[15] Zhang H, Vincent JL (1993). Arteriovenous differences in PCO2 and pH are good indicators of critical hypoperfusion. The American review of respiratory disease.

[16] Van der Linden P, Rausin I, Deltell A, Bekrar Y, Gilbart E, Bakker J, Vincent JL (1995). Detection of tissue hypoxia by arteriovenous gradient for PCO2 and pH in anesthetized dogs during progressive hemorrhage. Anesthesia and analgesia.

[17] Groeneveld AB, Vermeij CG, Thijs LG (1991). Arterial and mixed venous blood acid-base balance during hypoperfusion with incremental positive end-expiratory pressure in the pig. Anesthesia and analgesia.

[18] Vallet B, Teboul JL, Cain S, Curtis S (2000). Venoarterial CO(2) difference during regional ischemic or hypoxic hypoxia. J Appl Physiol (1985).

[19] Mallat J, Pepy F, Lemyze M, Gasan G, Vangrunderbeeck N, Tronchon L, Vallet B, Thevenin D (2014). Central venous-to-arterial carbon dioxide partial pressure difference in early resuscitation from septic shock: a prospective observational study. European journal of anaesthesiology.

[20] Ryoo SM, Lee J, Lee YS, Lee JH, Lim KS, Huh JW, Hong SB, Lim CM, Koh Y, Kim WY (2018). Lactate Level Versus Lactate Clearance for Predicting Mortality in Patients With Septic Shock Defined by Sepsis-3. Critical care medicine.

[21] Pope JV, Jones AE, Gaieski DF, Arnold RC, Trzeciak S, Shapiro NI, Emergency Medicine Shock Research Network I (2010). Multicenter study of central venous oxygen saturation (ScvO(2)) as a predictor of mortality in patients with sepsis. Ann Emerg Med.

[22] Teboul JL, Mercat A, Lenique F, Berton C, Richard C (1998). Value of the venous-arterial PCO2 gradient to reflect the oxygen supply to demand in humans: effects of dobutamine. Critical care medicine.

[23] Bakker J, Vincent JL, Gris P, Leon M, Coffernils M, Kahn RJ (1992). Veno-arterial carbon dioxide gradient in human septic shock. Chest.

[24] Kocsi S, Demeter G, Erces D, Nagy E, Kaszaki J, Molnar Z (2013). Central Venous-to-Arterial CO2 Gap Is a Useful Parameter in Monitoring Hypovolemia-Caused Altered Oxygen Balance: Animal Study. Critical care research and practice.

[25] Mallat J, Lemyze M, Meddour M, Pepy F, Gasan G, Barrailler S, Durville E, Temime J, Vangrunderbeeck N, Tronchon L (2016). Ratios of central venous-to-arterial carbon dioxide content or tension to arteriovenous oxygen content are better markers of global anaerobic metabolism than lactate in septic shock patients. Annals of intensive care.

[26] Mehl A, Harthug S, Lydersen S, Paulsen J, Asvold BO, Solligard E, Damas JK, Edna TH (2015). Prior statin use and 90-day mortality in Gram-negative and Gram-positive bloodstream infection: a prospective observational study. European journal of clinical microbiology & infectious diseases: official publication of the European Society of Clinical Microbiology.

[27] Munford RS (2006). Severe sepsis and septic shock: the role of gram-negative bacteremia. Annual review of pathology.

[28] Arora R, Campbell JP, Simon G, Sahni N (2017). Does serum procalcitonin aid in the diagnosis of bloodstream infection regardless of whether patients exhibit the systemic inflammatory response syndrome?. Infection.

[29] Thomas-Ruddel DO, Poidinger B, Kott M, Weiss M, Reinhart K, Bloos F, group Ms (2018). Influence of pathogen and focus of infection on procalcitonin values in sepsis patients with bacteremia or candidemia. Critical care.

[30] Hattori T, Nishiyama H, Kato H, Ikegami S, Nagayama M, Asami S, Usami M, Suzuki M, Murakami I, Minoshima M (2014). Clinical value of procalcitonin for patients with suspected bloodstream infection. American journal of clinical pathology.

[31] Povoa P, Coelho L, Almeida E, Fernandes A, Mealha R, Moreira P, Sabino H (2005). Pilot study evaluating C-reactive protein levels in the assessment of response to treatment of severe bloodstream infection. Clinical infectious diseases: an official publication of the Infectious Diseases Society of America.

[32] Lee YK, Hwang SY, Shin TG, Jo IJ, Suh GY, Jeon K (2016). Prognostic Value of Lactate and Central Venous Oxygen Saturation after Early Resuscitation in Sepsis Patients. PloS one.

[33] Mahmoodpoor A, Shadvar K, Sanaie S, Golzari SEJ, Parthvi R, Hamishehkar H, Nader ND (2020). Arterial vs venous lactate: Correlation and predictive value of mortality of patients with sepsis during early resuscitation phase. Journal of critical care.

[34] Wittayachamnankul B, Chentanakij B, Sruamsiri K, Chattipakorn N (2016). The role of central venous oxygen saturation, blood lactate, and central venous-to-arterial carbon dioxide partial pressure difference as a goal and prognosis of sepsis treatment. Journal of critical care.

